# Review of “Statistical Modelling of Molecular Descriptors in QSAR/QSPR” by Matthias Dehmer, Kurt Varmuza, and Danail Bonchev

**DOI:** 10.1186/1758-2946-4-36

**Published:** 2012-12-14

**Authors:** Alexandru T Balaban

**Affiliations:** 1 Department of Marine Sciences, Texas A&M University at Galveston, 200 Seawolf Parkway, Galveston, TX 77553, USA

## Book details

Statistical Modelling of Molecular Descriptors in QSAR/QSPR. Edited by Matthias Dehmer, Kurt Varmuza, and Danail Bonchev. Wiley-Blackwell, Weinheim, Germany, 2012. Print ISBN: 978-3-527-32434-7; ePDF ISBN: 978-3-527-64502-2. The book is Volume 2 of the series Quantitative and Network Biology, Series Editors: M. Dehmer and F. Emmert-Streib. Hard copy US$ 159.95, Euros 129.99; E-book US$ 126.00, Euros 114.00

The editors have selected 40 authors from 12 countries to contribute in producing a coherent account for the topics related to the book title. The 15 chapters of this book describe the current theoretical and practical results of statistical analysis and modeling of molecular descriptors. Zero-dimensional, 1-, 2-, and 3-dimensional descriptors are briefly mentioned. The most common 2D molecular descriptors are topological indices, and the use of any descriptor needs always a valid statistical confirmation that correlations are not due to chance. The first two chapters present, with some unavoidable overlap, introductory generalities about descriptors, various methods for modeling (multiple linear regression, partial least squares, neural networks, etc.) and for classification (linear discriminant analysis, k-nearest neighbor, support vector machine, etc.). Various software types are presented (e. g. Dragon, CODESSA, etc.).

The following chapters deal with more restricted aspects. In the 3^rd^ chapter, after once again the software packages for descriptor calculation are reviewed, the Mold^2^ freeware is presented in detail, with a long list of all 777 descriptors. A few examples are described: in comparison with results obtained for classification (by Cerius^2^, Dragon, and Molconn_Z) and regression models (by CODESSA), Mold^2^ is seen to provide equal or better QSAR results. The 4^th^ chapter is authored by V. Consonni and R. Todeschini, who enumerate in detail all relevant graph-theoretical indices and who describe with loading plots how the classes of matrices and indices contained in the Dragon software reflect the principal components.

In chapter 5, A. G. Mercader and E. A. Castro describe the use of partial-order ranking and linear modeling for predictive QSAR/QSPR studies. They cite arguments showing that the enhanced or modified replacement methods yield superior results when compared to genetic algorithms. The authors describe how to select descriptors for ranking and constructing Hasse diagrams in order to illustrate partial ordering.

Descriptors for branched polymers and their uses for predicting gyration ratios and chain dynamics are described with mathematical rigor in Chapter 6. Chapter 7 presents methods for evaluating quantitatively structural similarity Various clustering methods are also reviewed in this chapter by Irene Luque-Ruiz, G. Ceruela-Garcìa and M. A. Gómez-Nieto.

For compound database mining, Chapter 8 describes statistical methods for ligand-based virtual screening. In improving bioactive lead compound structures, the Kullbach-Leibler divergence is used for quantifying the differences in value distributions of active and inactive compounds. Bayesian screening is exemplified in a recall prediction approach using the MACCS fingerprint.

In this reviewer’s opinion, the 9^th^ chapter on molecular descriptors and electronic structure is too long (48 pages). For instance, it presents on ten pages with half-page color plots trivial properties of the five hexane isomers, or it uses another half-page plot in order to prove lack of correlation, and fails to convey the more general and comprehensive aspects covered by the chapter’s title.

By contrast, Chapter 10 by C. Kramer and T. Clark (12 pages) should have been longer because it presents new types of descriptors and models in SQAR/QSPR that are less familiar. Non-covalent molecular interactions are often crucial for biologically active structures. Molecular electrostatic potential (MEP) may be calculated by the atoms-in-molecules approach. Electron densities, local polarizabilities, local ionization energies, and local electron affinities can be calculated by density functional theory. Property-encoded surface translator (PEST) descriptors represent properties on 3D surfaces in a compact way. Hydrogen-bonding strengths are reflected in MEP. The comparative molecular field analysis and comparative molecular similarity analysis (CoMFA and CoMSIA, respectively) compute interaction energies in 3D grid points and then proceed by using a partial least squares fitting algorithm for pharmacophore models. T. Clark published in 2010 a surface integral model for the octanol-water distribution coefficient logP_ow_. With large pools of descriptors, validation methods are essential and it appears that a new approximation of critical Fisher values for multiple linear regressions solves this problem.

Chapter 11 is entitled “Consensus Models of Activity Landscapes”. If the structure-activity correlation for a set of molecules is represented by a 3D plot in which the x, y axes correspond to two principal components and the biological activity is along the z axis, then this landscape may have *activity cliffs* when compounds with similar structure have unexpectedly different activities. Computer programs analyzing activity landscapes are expertly described in this chapter. Consensus models have been developed using structure-activity similarity maps and related graph representations.

Chapter 12 presents a study for discovering chemical reaction networks from the experimentally determined concentrations versus time. Topological relationships between reacting species can be deduced by using reverse engineering for finding the differential equations compatible with observed reaction dynamics.

In Chapter 13 *ab*-*initio* quantum chemical methods are tested for explaining properties of a simple molecule with low symmetry (hydrogen peroxide). The current methods are compared, showing that self-consistent field calculations are the least accurate, and the singles & doubles coupled cluster via a perturbational treatment are the most accurate.

In Chapter 14, D. J. Graham provides an analysis of organic reaction pathways by Brownian processing of hydrogen-depleted molecular graphs. On interpreting chemical reactions of organic molecules as changes in two coordinates (mutual information and dispersion energy for electronic messages, reduced in terms of constituent ethane units) one can calculate distances between molecules (state points).

In the last chapter, an up-to-date account of computer-assisted organic synthesis is presented by G. Novak and G. Fic. With millions of reactions stored in the Beilstein and CASREACT databases, and the numerous programs for applying graph theoretical approaches for retrosynthetic disconnections, this chapter provides excellent information about past and present progress in this field.

A few final remarks follow. One sees at present a kind of epidemics in which authors use too many acronyms that slow down the reading, especially if there is no alphabetical list of such acronyms. For most chemists, TFA means trifluoroacetic acid, but in chapter 12 it means target factor analysis. A text does not look more “scientific” if it abounds in such abbreviations! It is unfortunate that several chapters in this book suffer from this deficiency. A positive aspect is that most chapters have bibliographies that include paper titles, which help the reader in deciding whether to look up the corresponding reference. Graphic conditions (print, color illustrations, format of figures and tables) are excellent.

## Competing interests

The author declares that he has no competing interests.

